# The Phlorotannin-Rich Fraction of *Ecklonia cava* Extract Attenuated the Expressions of the Markers Related with Inflammation and Leptin Resistance in Adipose Tissue

**DOI:** 10.1155/2020/9142134

**Published:** 2020-03-07

**Authors:** Myeongjoo Son, Seyeon Oh, Junwon Choi, Ji Tae Jang, Chang Hu Choi, Kook Yang Park, Kuk Hui Son, Kyunghee Byun

**Affiliations:** ^1^Department of Anatomy & Cell Biology, Gachon University College of Medicine, Incheon 21936, Republic of Korea; ^2^Functional Cellular Networks Laboratory, College of Medicine, Department of Medicine, Graduate School and Lee Gil Ya Cancer and Diabetes Institute, Gachon University, Incheon 21999, Republic of Korea; ^3^Aqua Green Technology Co., Ltd., Smart Bldg., Jeju Science Park, Jeju 63309, Republic of Korea; ^4^Department of Thoracic and Cardiovascular Surgery, Gachon University Gil Medical Center, Gachon University, Incheon 21565, Republic of Korea

## Abstract

Obesity is associated with systemic chronic inflammation, and it induces central leptin resistance which blocks the appetite-suppressing effect of leptin and leptin resistance in adipocytes. In the present study, we evaluated the effects of *Ecklonia cava* extract (ECE), which contained rich phlorotannins, on inflammation and leptin resistance in the adipose tissue of a diet-induced obese model. Effects of ECE on fat deposition, inflammation, M1/M2 macrophage, and T-cell infiltrations were investigated, and leptin resistance and SOCS3 were also measured in adipose tissue. Furthermore, ECE attenuated the expression of inflammation-related receptors such as TLR4 and RAGE and leptin resistance by reducing SOCS3 expression, increasing expression of leptin receptor in adipose tissue, and increasing lipolysis. ECE showed antiadiposity and anti-inflammatory effects, attenuated leptin resistance, and increased lipolysis in the diet-induced obese model. This study shows that ECE is a suitable dietary supplement candidate for the prevention or treatment of obesity or obesity-associated diseases, especially inflammation-related diseases.

## 1. Introduction

Obesity leads to chronic inflammation and changes the secretion patterns of adipokines in adipose tissue. In obese fat tissue, hypertrophic adipocytes express and secrete high levels of proinflammatory cytokines, such as tumor necrosis factor *α* (TNF-*α*), interleukin (IL)-6, IL-8, and monocyte chemoattractant protein-1 (MCP-1) [1, 2]. These cytokines also promote and aggravate adipose tissue inflammation by recruiting immune cells, including macrophages and T cells [2, 3]. Low-grade chronic inflammation in adipose tissue is attracting increasing attention because it is viewed to underlie obesity-related complications [4], such as diabetes, coronary heart diseases, hyperlipidemia, hypertension, and cancer [5].

Leptin is perhaps the most well-known adipokine and is produced by adipocytes. Leptin regulates food intake and energy expenditure by interacting with hypothalamic nuclei and plays a crucial role in the maintenance of body weight [6]. In fact, circulating leptin levels are proportional to body fat, but in diet-induced obesity, these increased levels fail to prevent weight gain. This unresponsiveness to endogenous (or exogenous) leptin is referred to as “leptin resistance” [7, 8]. Leptin acts by binding to functional long isoform leptin receptor (ObR) in the central nervous system (CNS), especially in the hypothalamus, and ObR is known to activate Janus-activating kinase 2-signal transducer/activator of transcription 3 (JAK2/STAT3) and phosphatidylinositol 3-kinase (PI3K) signaling pathways [9, 10]. The ability of leptin in activating STAT3 and PI3K is diminished in diet-induced obese mice [10–12] and leptin increased expression of suppressor of cytokine signaling-3 (SOCS3) which is a main inducer of leptin resistance in the CNS. Furthermore, SOCS3 acts as a potent negative regulator of the JAK/STAT pathway and thus constitutes a negative feedback loop [13, 14]. SOCS3 is also involved in the onset of leptin resistance at the central and peripheral levels [15]. Obesity promotes SOCS3 expression in the hypothalamus [14, 16], adipose tissue [16, 17], liver [16, 18], and skeletal muscle [16, 19], and SOCS3 might also upregulate the expressions of adipocyte inflammatory cytokines like IL-6, TNF-*α*, and MCP-1 [20]. These results indicate that leptin resistance in adipocytes might influence adipocyte inflammation.

*Ecklonia cava* (*E*. *cava*) is a species of brown alga abundant in the neritic regions of Korea and Japan [21, 22] and has attracted research because of the medicinal effects of its components, which include carotenoids, fucoidans, and phlorotannins [21–26], and its anti-inflammatory, antioxidative, antidiabetic [27, 28], and antiobesity effects [21]. It has been reported that the *E*. *cava* extract (ECE) reduces body weight gain, body fat, and hyperglycemia, improves glucose tolerance due to its anti-inflammatory effects, and enhances lipid metabolism in a high-fat diet-induced (HFD-induced) animal model of obesity [21]. Although its anti-inflammatory effects are well-established, little is known of its effects on inflammation and leptin resistance in adipose tissue relatively.

*Garcinia cambogia* is a tropical edible fruit of the family Clusiaceae and is grown in south East Asia, southern India, and Africa [8, 29]. The dried fruit rinds of *Garcinia cambogia* contain 20–30% by weight of (-) hydroxycitric acid (HCA) [9], which is a competitive inhibitor of ATP-citrate lyase, a key enzyme involved in fatty acid and cholesterol biosynthesis [8, 30]. Many studies have investigated the antiobesity effect of the *Garcinia cambogia* extract (GCE). For example, supplementation of HFD with GCE significantly reduced body weight gain, glucose intolerance, and plasma leptin and TNF-*α* levels in the obese mouse model [8]. In the present study, we evaluate the effects of ECE on inflammation and leptin resistance in adipose tissue in a HFD-induced obese animal model and compared its effects with those of GCE.

## 2. Materials and Methods

### 2.1. Preparations of ECE and GCE

The ECE was manufactured as follows. *E. cava* collected near the Jeju coast was washed thoroughly and air dried for 48 hr at room temperature. The *E. cava* was extracted and finely grounded using 50% aqueous ethanol for 12 hr at 85°C. The *E. cava* extract was filtered, concentrated, and sterilized (40 to 60 min at 85°C). Finally, a dry powder from the *E. cava* extract was produced by spray-drying. Phlorotannins of the prepared ECE was validated using HPLC ([Supplementary-material supplementary-material-1]). GCE was obtained from ESFood (Lot. 0831; Gunpo, Republic of Korea), and the GCE containing 60% HCA was used as a positive control.

### 2.2. Animal Study Design

Male mice C57BL/6N (7 weeks old) were purchased from Orient Bio (Republic of Korea) and maintained under controlled conditions (temperature is 23°C and 50% humidity under 12 hr dark/light cycle). After seven days of acclimatization, mice were randomly divided into four groups in which (1) mice were fed a regular chow diet for 4 weeks and then orally administered 0.9% normal saline for 4 weeks (the NFD group) and (2) mice were fed a 45% high-fat diet (HFD; Research Diet Inc., USA) for 4 weeks and then orally coadministered 0.9% normal saline (the HFD/saline group), ECE (70 mg/kg/day; the HFD/ECE group), or GCE (500 mg/kg/day; the HFD/GCE group) for 4 weeks. After oral administration, visceral fat samples were collected from retroperitoneal adipose tissue in accordance with the ethical principles issued by the Institutional Animal Care and Use Committee of Gachon University (approval number: LCDI-2017-0034). Body weights and food intakes were measured weekly, and body masses of fat and lean mice were measured using the 1Hminispec system (Bruker Optik, LF90II; Germany).

### 2.3. RNA Extraction and Quantitative Real-Time Polymerase Chain Reaction (qRT-PCR)

The visceral fat tissues (200 mg) were homogenized by using gentleMACS *M* tubes (MiltenyiBiotec, Germany) in 500 *μ*l of RNiso (TAKARA, Japan). Homogenates were then added to 0.1 ml of chloroform, mixed, and centrifuged at 12,000 ×g for 15 min at 4°C. Aqueous phases were collected, placed in cleaned tubes, mixed with 0.25 ml of isopropanol, and centrifuged using the same conditions. Isolated RNA samples were then washed with 0.5 ml 70% ethanol and dissolved in 30 *μ*l of diethyl pyrocarbonate- (DEPC-) treated water. For qRT-PCR (quantitative real-time polymerase chain reaction), cDNA was synthesized from 1 *μ*g of total RNA using a Prime Script 1st strand cDNA Synthesis Kit (TAKARA, Japan). Synthesized cDNA was used as a template and qRT-PCR was performed using the CFX 384 Touch TM Real-Time PCR detection system. Reaction efficiencies and threshold cycle numbers were determined using CFX Manager TM Software. Primer sequences for target genes are detailed in [Supplementary-material supplementary-material-1].

### 2.4. Paraffin Block Preparation

Visceral fat was harvested from retroperitoneal visceral fat. The fat tissues were fixed in 4% paraformaldehyde (Biosesang, Republic of Korea) overnight at 4°C and placed in an automatic dehydration machine (Leica, UK). Tissues were dehydrated in a series of steps, that is, with 90% ethanol 3 times for 1 hr followed by 100% ethanol 2 times for 2 hr and then cleared with 100% xylene 3 times for 1.5 hr and embedded in paraffin.

### 2.5. Histologic Staining and Quantification

For visceral fat size measurements, visceral fat paraffin tissues were sectioned at 7 *μ*m and stained with hematoxylin (DAKO, Japan) and eosin (Sigma-Aldrich, USA). The stained images were obtained using an Axio Imager Z1 upright microscopy system (Carl Zeiss, Germany). Adipocyte areas were determined from the cross-sectional areas of adipocyte cell membranes using Image J software (NIH, USA).

### 2.6. Immunostaining

Visceral fat tissue sections were incubated with anti-TLR4 (dilution rate 1 : 100), anti-RAGE (dilution rate 1 : 100), anti-SOCS3 (dilution rate 1 : 100), and anti-ObR (dilution rate 1 : 100) antibodies for 1 day at 4°C, rinsed twice with phosphate-buffered saline (PBS), incubated with fluorescent conjugated secondary antibodies for 1 hr in blocking solution, and rinsed twice with PBS. To detect nuclei of fat tissue, DAPI (Sigma-Aldrich, USA) was used for 3 min. The fluorescent images were detected with confocal microscopy (LSM 710; Carl Zeiss, Germany), and quantification of the intensity of the fluorescent was detected using ZEN software 2012 version (Carl Zeiss, Germany).

### 2.7. ELISA for Serum Leptin and Adiponectin

Serum samples were obtained by incubating collected blood at room temperature for 20 min and centrifuging at 2,000 ×g for 10 min at 4°C. Absolute concentrations of adipokines like leptin (Invitrogen, USA) and adiponectin (Abcam, UK) in serum were determined using ELISA kits. Absorbances were measured at 450 nm using an ELISA plate reader (Molecular Devices, USA).

### 2.8. Statistical Analysis

The Kruskal–Wallis test and the Mann–Whitney *U* test as a post hoc test were used to determine the significances of differences among the NFD/saline, HFD/saline, HFD/ECE, and HFD/GCE groups. Results are presented as mean ± SD, and statistical significance was accepted for *p* values < 0.05. The analysis was performed using SPSS version 22 (IBM Corporation, USA).

## 3. Results and Discussion

### 3.1. Attenuating Effects of *E*. *cava* Extract on HFD-Induced Adiposity, Increased Food Intake, and Adipocyte Size

**Figure 1:**
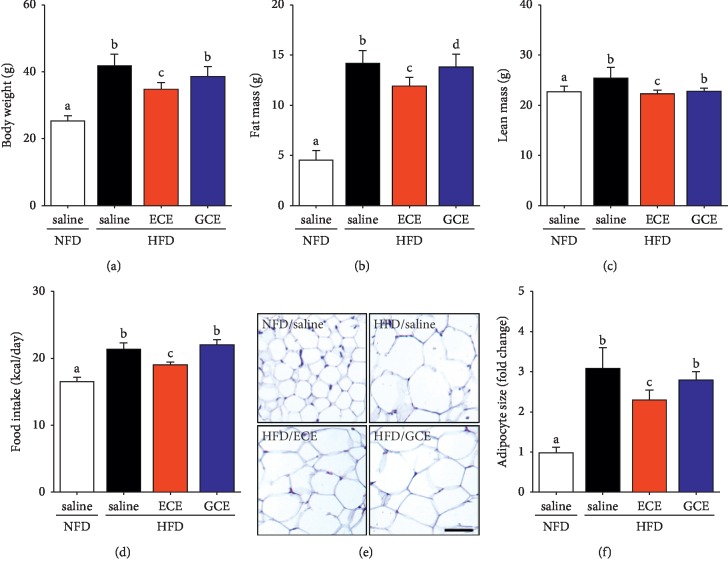
Effects of *E*. *cava* extract on body weight, adiposity, food intake, and adipocyte sizes in high-fat diet- (HFD-) induced obese mice. Mice fed with standard normal fat diet (NFD; open bar) or HFD (HFD; black bar) for 4 weeks and then 0.9% saline (NFD/saline group and HFD/saline group), 70 mg/kg/day *E*. *cava* extract (HFD/ECE group; red bar) or 500 mg/kg/day *Garcinia cambogia* extract (HFD/GCE group; blue bar) orally administrated for the next 4 weeks. After 8 weeks of administration, (a) body weight, (b) fat mass, (c) lean mass, and (d) food intake were measured. (e) Hematoxylin and eosin (H&E) stained images showing adipocytes in visceral fat and (f) a graph showing average visceral adipocyte size in representative H&E stained images. Adipocyte size was expressed as folds of that of NFD/saline group. Scale bar = 200 *μ*m. The same letters represent no significant difference (*p* < 0.05).

ECE attenuated HFD-induced body weight and fat mass gain more than GCE. Animals in the HFD/saline group gained significantly more weight than animals in the NFD/saline group (Figure 1(a)), and animals in the HFD/ECE group gained significantly less weight than animals in the HFD/saline group. However, weight gain in the HFD/GCE group was not significant different from that in the HFD/saline group. Fat mass was significantly greater in the HFD/saline group than in the NFD/saline group. The fat mass and lean mass of the HFD/ECE group were significantly decreased than in the HFD/saline group, and they were significantly different in the HFD/ECE and HFD/GCE groups (Figures 1(b) and 1(c)). The lean mass was decreased significantly in the HFD/ECE group than that in the HFD/saline group, but the decrease was not significant in the HFD/GCE group than in the HFD/saline group (Figure 1(c)). In addition, ECE decreased food intake more than GCE, and food intake was higher in the HFD/saline group than in the NFD/saline group but lower in the HFD/ECE group than in the HFD/saline group. However, food intakes in the HFD/GCE and HFD/saline groups were nonsignificantly different (Figure 1(d)). Furthermore, the mean white adipocyte sizes in visceral fat tissues in the HFD/saline, HFD/ECE, and HFD/GCE groups were greater than in the NFD/saline group and were significantly smaller in the HFD/ECE group than in the HFD/saline group (Figure 1(e)).

### 3.2. Effects of *E*. *cava* Extract on Activated Macrophage Infiltration, M1 Polarization, and T-Cell Infiltration in Adipose Tissue

**Figure 2:**
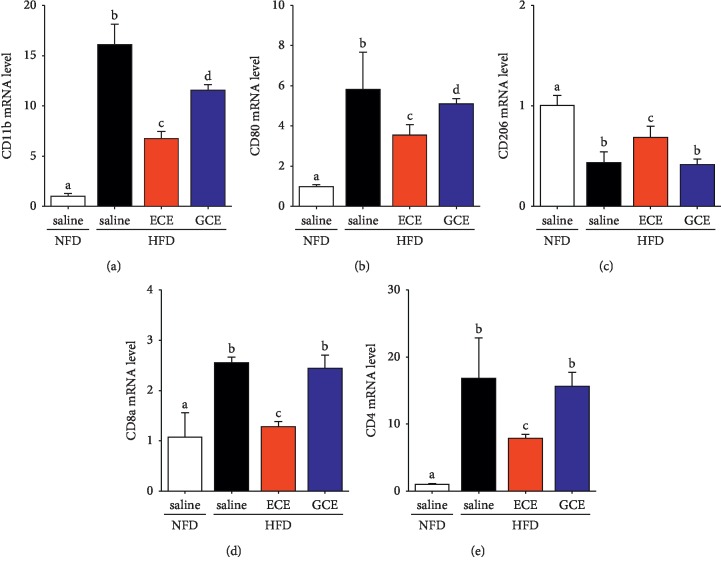
Effects of *E*. *cava* extract on macrophages and T-cell infiltration in visceral fat of high-fat diet- (HFD-) induced obese mice. (a) CD11b as a marker of total macrophage; (b) CD80 as a marker of M1 macrophage; and (c) CD206 as a marker of M2 macrophage expression mRNA levels were measured by qRT-PCR in visceral fat of standard normal fat diet with 0.9% saline (NFD/saline; open bar), HFD with 0.9% saline (HFD/saline; black bar), HFD with *E*. *cava* extract (HFD/ECE; red bar), and HFD with *Garcinia cambogia* extract (HFD/GCE; blue bar) groups. (d, e) CD8a and CD4 as T-cell expression levels were determined by qRT-PCR in visceral fat of the same groups. All mRNA levels expressed relative level and normalized *ß*-actin of the NFD/saline group. The same letters represent no significant difference (*p* < 0.05).

The expression of CD11b (an activated macrophage marker) [31] mRNA in visceral fat was significantly higher in the HFD/saline group than in the NFD/saline group and significantly lower in the HFD/ECE and HFD/GCE groups than in the HFD/saline group. The CD11b expression was significantly lower in the HFD/ECE group than in the HFD/GCE group (Figure 2(a)). CD80 expression (a M1 macrophage marker) [32, 33] was significantly lower in the HFD/ECE and HFD/GCE groups than in the HFD/saline group. The CD80 expression in HFD/ECE was significantly lower than that in the HFD/GCE group. The CD206 (marker of M2) [32, 33] was significantly higher in the HFD/ECE group than in the HFD/saline group. The total number of macrophages in adipose tissue, which were classified as M1 (classically activated) and M2 phenotypes (alternatively activated) [2, 34], prominently increases with obesity. The M1 type prominently expresses inducible nitric oxide synthase (iNOS), TNF-*α*, IL-1*β*, and CD11c on cell surfaces, whereas the M2 type topically expresses arginase, IL-10, and Ym-1 [2]. It has been established that obesity induces the polarization of macrophages in fat tissues from the M2 to the M1 phenotype [35], and it has been reported that M1 macrophages are the major cause of adipose tissue inflammation and insulin resistance in obesity [2]. In the present study, ECE attenuated HFD-induced macrophage infiltration into fat tissues and M1 polarization more effectively than GCE (Figures 2(b) and 2(c)). The number of Th1, a subtype of CD4, and CD8 T cells is elevated in adipose tissues of obese subjects [2, 36–38]. The Th1 cell and CD 8 T cell are also major interferon gamma- (IFNγ-) expressing cells that accumulate in obese people [38]. IFN*γ* stimulates the expression of chemokines and proinflammatory cytokines in adipocytes as well as the M1 polarization of macrophages [2]. In visceral fat tissue, the expressions of CD8a and CD4 mRNAs in T cells were significantly higher in the HFD/saline group than in the NFD/saline group, and these expressions were significantly lower in the HFD/ECE group than in the HFD/saline group. ECE attenuated HFD-induced T-cell expression significantly higher than GCE (Figures 2(d) and 2(e)). Like visceral fat tissues, the ECE and pyrogallol-phloroglucinol-6,6-bieckol (PPB), a representative phlorotannin from ECE, attenuate endothelial cell dysfunction by controlling perivascular fat tissue dysfunction through decreasing inflammation and ER stress and modulating brown adipocyte function [39].

### 3.3. Effects of *E*. *cava* Extract on Inflammatory Cytokine Production in Fat Tissue

**Figure 3:**
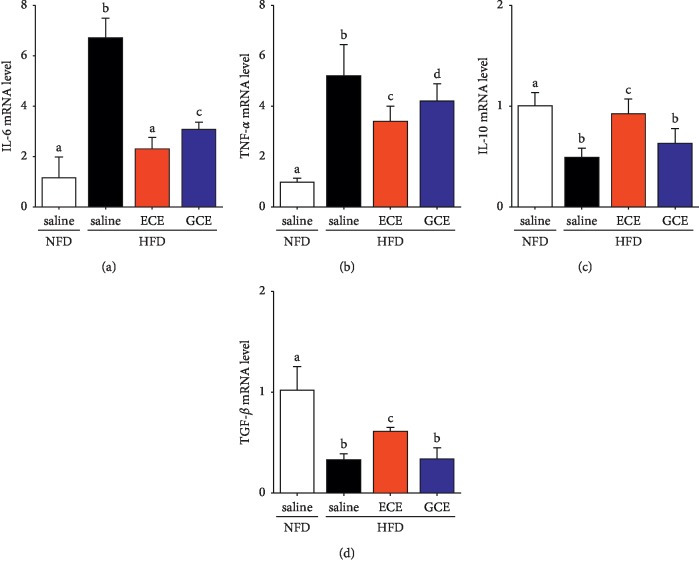
Effects of *E*. *cava* extract on inflammatory cytokines expression in visceral fat on high-fat diet- (HFD-) induced obese mice. (a, b) IL-6 and TNF-*α* as M1 macrophage-related inflammatory cytokines and (c, d) IL-10 and TGF-*ß* as M2 macrophage-related cytokines in visceral fat of standard normal fat diet with 0.9% saline (NFD/saline; open bar), HFD with 0.9% saline (HFD/saline; black bar), HFD with *E*. *cava* extract (HFD/ECE; red bar), and HFD with *Garcinia cambogia* extract (HFD/GCE; blue bar) group. All expression levels were determined by qRT-PCR, and all mRNA levels expressed relative level and normalized *ß*-actin of the NFD/saline group. The same letters represent no significant difference (*p* < 0.05).

Obesity-induced increases in M1 macrophage infiltration [40], T-cell numbers, and hypertrophic adipocyte numbers [2] have been reported to enhance proinflammatory response in adipose tissue and to increase secretions of inflammatory cytokines (e.g., TNF-*α* and IL-6) and decrease secretion of anti-inflammatory cytokine (e.g., IL-10 or TGF-*β*) [40–42]. We observed that the expressions of IL-6 and TNF-*α* mRNA were significantly higher in the HFD/saline group than in the NFD/saline group. The inflammatory cytokines were decreased in both of the HFD/ECE and HFD/GCE groups, and the decreasing effect of HFD/ECE group was more prominent than that of the HFD/GCE group (Figures 3(a) and 3(b)). In addition, the expressions of IL-10 and TGF-*β* mRNA were significantly lower in the HFD/saline group than in the NFD/saline group. These levels were increased in the HFD/ECE group, and these levels in the HFD/ECE group were greater than those in the HFD/GCE group (Figures 3(c) and 3(d)).

### 3.4. Effects of *E*. *cava* Extract on TLR4, RAGE, and NF-*κ*B Protein Expressions in Adipose Tissue

**Figure 4:**
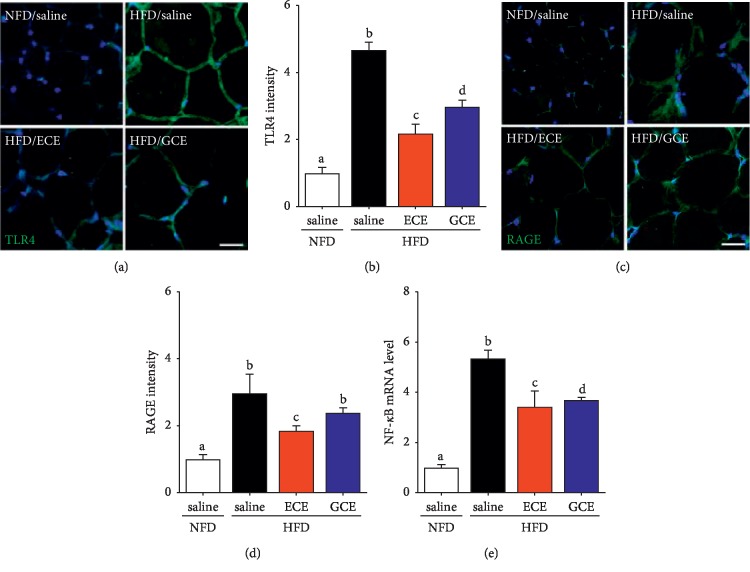
Effects of *E*. *cava* extract on TLR4, RAGE, and NF-κB expressions in visceral fat of high-fat diet- (HFD-) induced obese mice. To validate expression of inflammation-related receptors in visceral fat of standard normal fat diet with 0.9% saline (NFD/saline; open bar), HFD with 0.9% saline (HFD/saline; black bar), HFD with *E*. *cava* extract (HFD/ECE; red bar), and HFD with *Garcinia cambogia* extract (HFD/GCE; blue bar) group, tissue section slides were prepared. (a, c) The confocal microscopic images show toll-like receptor 4 (TLR4; (a) green), the receptor of AGE (RAGE; (c) green) expressions were assessed by immunostaining, and nuclei were stained DAPI (blue). (b, d) Quantitative green fluorescence intensity graphs show folds of those of the NFD/saline group, and these graphs show average expression levels in representative images. (e) NF-*κ*B expressions were assessed by qRT-PCR. Scale bar = 50 *μ*m. The same letters represent no significant difference (*p* < 0.05).

The productions of inflammatory cytokines, such as TNF-*α*, IL-6, and IL-1*β*, are induced by TLR4 signaling [43, 44]. In one *in vitro* study, it was shown that ECE acts as a negative regulator of inflammation by suppressing TLR4 signaling and the secretions of TNF-*α*, IL-6, IL-1*β*, and IL-10 by lipopolysaccharide-stimulated macrophages [43]. In our study, ECE attenuated TLR4 expression in visceral adipose tissue (Figures 4(a) and 4(b)). Advanced glycation end products (AGEs), members of the proinflammatory S100/calgranulin family, and high motility group box 1 protein are the ligands which bind to the receptor for advanced glycation end products (RAGE) [45, 46]. Inflammation-related signaling cascades involving nuclear factor (NF)-κB (Figures 4(e) and [Supplementary-material supplementary-material-1]), extracellular signal-regulated kinase (ERK) 1/2, mitogen-activated protein kinases (p38 MAPK), c-Jun N-terminal kinases (JNK), protein kinase C (PKC), Rac/Cdc42, or TIR-domain containing adapter protein (TIRAP) and MyD88 (adapter proteins for TLR2 and 4) are activated by binding RAGE and its ligands [45, 46]. These observations regarding the potential regulatory role of RAGE in inflammation suggest that RAGE might importantly contribute to inflammation and to the obesity-associated dysregulation of adipokines [47]. Several studies have shown that RAGE is related to obesity. Especially, the adipocytes of obese human show higher RAGE expression than those of the lean human [47] and accumulate RAGE ligands as well in the adipose tissues of HFD and obesity models [48–50]. In our study, ECE reduced TLR4, RAGE, and NF-*κ*B expressions in the adipose tissues of HFD-fed mice (Figures 4(c) and 4(d)).

### 3.5. Effects of *E*. *cava* Extract on Serum Leptin and Adiponectin Levels and on Leptin Resistance in Adipose Tissue

**Figure 5:**
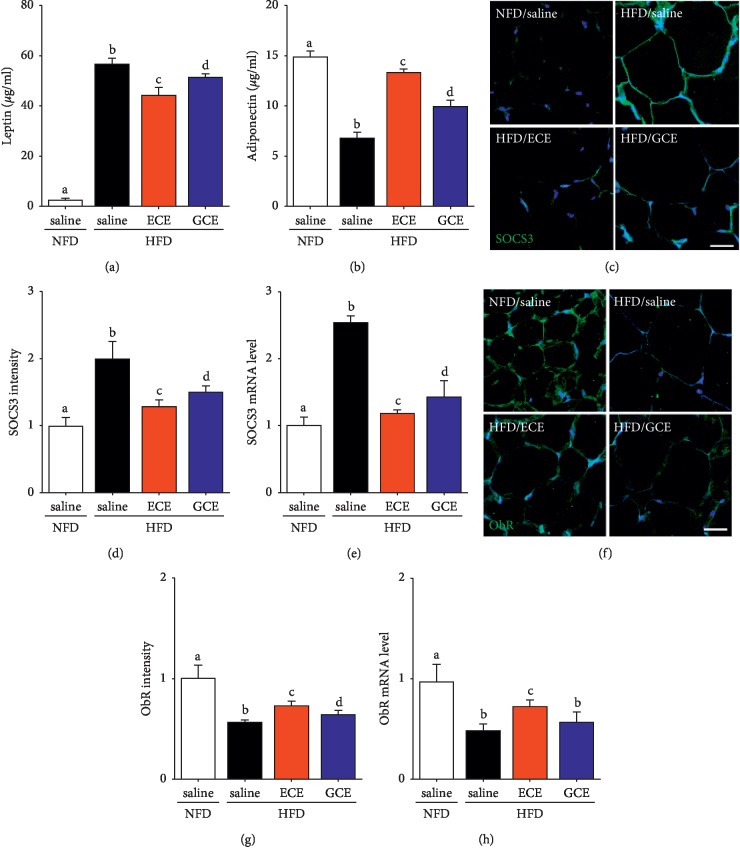
Effects of *E*. *cava* extract on serum leptin and adiponectin levels and on leptin resistance in visceral fat of high-fat diet- (HFD-) induced obese mice. (a, b) Serum leptin and adiponectin levels were quantified by ELISA in standard normal fat diet with 0.9% saline (NFD/saline; open bar), HFD with 0.9% saline (HFD/saline; black bar), HFD with *E*. *cava* extract (HFD/ECE; red bar), and HFD with *Garcinia cambogia* extract (HFD/GCE; blue bar) groups. The suppressor of cytokine signaling 3 (SOCS3) as leptin resistance-related molecule protein level (c) (green), (d) quantified intensity graph, and the (e) SOCS3 mRNA expression level was validated by immunostaining and qRT-PCR. Like SOCS3, ObR receptor protein level ((f) green), (g) quantified graph, and (h) the ObR mRNA expression level were validated by immunostaining and qRT-PCR in visceral fat. Nuclei were detected by DAPI (blue) in confocal microscopic images. All mRNA levels expressed relative mRNA level and normalized ß-actin of the NFD/saline group. Scale bar = 50 *μ*m. The same letters represent no significant difference (*p* < 0.05).

Adipokine production is altered as adipose tissue mass increases. Most notably, adiponectin production drops and results in reduced glucose uptake and anti-inflammatory regulation of the local environment [22] and adipocytes start producing more leptin [28]. In our study, HFD caused decrease in adiponectin levels, and ECE attenuated this decrease more so than GCE. Also, HFD-induced leptin increases were reduced more by ECE than GCE (Figures 5(a) and 5(b)). Wang et al. proposed that white adipose tissue develops local leptin resistance, which may partially explain why circulating high concentrations of leptin in obese animals show little effect on white adipose tissue mass as well as the development of central leptin resistance in the central nerve system (CNS) [51, 52]. They also reported that activation of STAT3 was diminished in adipose tissue from leptin-resistant diet-induced obese rats under basal and leptin-stimulated conditions [51, 53]. Leptin resistance is associated with an early increase in the expression of suppressor of SOCS1 and 3, which inhibits JAK-STAT3 activation [14] and subsequently ObR expression [53]. Similarly, it has been reported that leptin receptor expression is greatly reduced in the adipose tissues of morbidly obese women [54]. We observed that SOCS3 protein and mRNA expression were elevated by HFD but diminished by ECE, which suggests HFD induces leptin resistance in fat tissue by upregulating SOCS3 expression (Figures 5(c)–5(e)). In addition, leptin receptor expression was decreased by HFD and this decrease was increased by ECE. Decreased ObR protein and mRNA expression and localization on adipocyte surfaces are key determinants of cell sensitivity to leptin [15]. Interestingly, PPB also restored leptin sensitivity in HFD-induced obese mice and ob/ob mice [55]. We also found that ECE inhibited HFD-induced decreases in ObR expression, which suggests that ECE enhanced adipocyte leptin sensitivity by maintaining ObR expression (Figures 5(f)–5(h)).

The HFD-induced condition might be ameliorated by *E. cava* which plays a direct effect on adipose tissue. The administration of *E. cava* (ECE) suppresses the increasing HFD-induced fat mass and adipocyte size by reducing the food intake, whereas neither the administration of saline nor GCE. Therefore, a possibility can be assured that less mRNA expression of leptin and inflammatory marker in ECE rats might be associated with less fat mass and adipocyte size in ECE compared to the saline and GCE groups.

### 3.6. Effects of *E*. *cava* Extract on Lipolysis in Adipocytes

**Figure 6:**
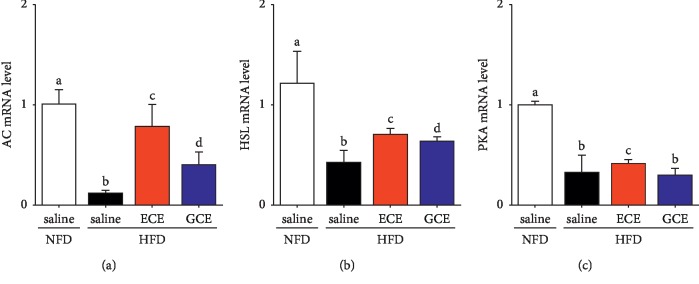
Effects of *E*. *cava* extract on adipocyte lipolysis in response to an agonist upregulation in visceral fat of high-fat diet- (HFD-) induced obese mice. To validate adipocyte lipolysis related markers such as (a) AC, (b) HSL, and (c) PKA in visceral fat of standard normal fat diet with 0.9% saline (NFD/saline; open bar), HFD with 0.9% saline (HFD/saline; black bar), HFD with *E*. *cava* extract (HFD/ECE; red bar), and HFD with *Garcinia cambogia* extract (HFD/GCE; blue bar) groups, these mRNA expression levels were assessed by qRT-PCR. All mRNA levels expressed relative level and normalized ß-actin of the NFD/saline group. The same letters represent no significant difference (*p* < 0.05).

In vitro, leptin decreases lipogenesis and increases triglyceride hydrolysis and fatty acid oxidation [56, 57]. However, in vivo, the effects of leptin on adipose tissue may be mediated through the peripheral nervous system, and leptin has been reported to increase sympathetic efferent signals to white adipose tissue and thus increase lipolysis [56, 58]. To initiate lipolysis, the activation of G*α*s leads to the activation of adenylyl cyclase (AC), the production of cAMP, which phosphorylates and activates cAMP-dependent protein kinase A (PKA) [59, 60], and the phosphorylation of hormone-sensitive lipase (HSL). Phosphorylated HSL is then translocated from the cytoplasm to the surfaces of lipid droplets and activates the hydrolysis of TG in adipocytes [61, 62]. Furthermore, it has been suggested that low HSL levels in adipose tissues from obese subjects contribute to the impairment of catecholamine-mediated lipolysis through a postreceptor defect [63]. In our study, the expressions of AC, HSL, and PKA were lower in the HFD/saline group than in the NFD/saline group but significantly higher in the HFD/ECE group than in the HFD/saline group, which suggested that ECE increased adipocyte lipolysis in response to an agonist by leptin in adipocytes (Figure 6).

ECE attenuated leptin resistance by reducing SOCS3 expression and increased leptin sensitivity by increasing leptin receptor expression in adipose tissues, and these modulations increased lipolysis in adipocytes and possibly reduced adipocyte sizes. In addition, it was previously found that the forced expression of SOCS3 increased the expressions of IL-6, TNF-*α*, and MCP-1 but decreased leptin expressions in 3T3-L1 adipocytes [20]. These observations suggest that SOCS3 promoted 3T3-L1 adipocyte inflammation by promoting the synthesis of inflammatory cytokines and inhibiting leptin signaling [20] and that the ECE induced downregulation of SOCS3 expression might reduce the activities of inflammatory cytokines in adipocytes, which in turn suggests controlling inflammation in fat tissues is important as this inflammation is the main pathophysiology underlying many obesity-related diseases.

In the present study, ECE significantly attenuated body weight, food intake, and adipocyte size increases in HFD-fed mice. Adipocyte hypertrophy creates proinflammatory conditions in fat tissues and leads to macrophage infiltration, M1 polarization, and the activation of CD4 and CD8 T cells, and these immune cells are the primary producers of the proinflammatory cytokines that induce inflammation in fat tissues. ECE administration decreased HFD-induced adiposity and attenuated inflammation in fat tissues by modulating macrophage and T-cell functions and reducing proinflammatory cytokine production and it also attenuated the expressions of TLR4 and RAGE, which might also induce inflammation in adipose tissue. In addition, ECE attenuated leptin resistance in adipose tissue, increased lipolysis, and reduced adipocyte sizes.

## 4. Conclusions

This study shows that *E*. *cava* extract (ECE) conferred antiadiposity, anti-inflammatory, and antileptin resistance effects and increased lipolysis in adipocytes. Our results suggest that ECE is a candidate for dietary supplement that inhibits the development of obesity and obesity-associated diseases, especially inflammation-related diseases.

## Data Availability

The data used to support the findings of this study are available from the corresponding author upon request.

## Supplementary Materials

Supplementary Materials Figure S1. High-performance liquid chromatography (HPLC) of *E. cava* extract. Figure S2. Effects of *E. cava* extract on NF-*κ*B expression in visceral fat of high-fat diet- (HFD-) induced obese mice. Table S1. List of primers for quantitative polymerase chain reaction (qRT-PCR) and supplementary materials and methods.
