# Supplementary material for: The Phlorotannin-Rich Fraction of Ecklonia cava Extract Attenuated the Expressions of the Markers Related with Inflammation and Leptin Resistance in Adipose Tissue
Source: Int J Endocrinol. 2020 Mar 7;2020:9142134. doi: 10.1155/2020/9142134 (PMC7081028; doi:10.1155/2020/9142134)
Supplement: Supplementary Materials — Figure S1. High-performance liquid chromatography (HPLC) of E. cava extract. Figure S2. Effects of E. cava extract on NF-κB expression in visceral fat of high-fat diet- (HFD-) induced obese mice. Table S1. List of primers for quantitative polymerase chain reaction (qRT-PCR) and supplementary materials and methods. [file 9142134.f1.docx]

**Supplementary figure**

**
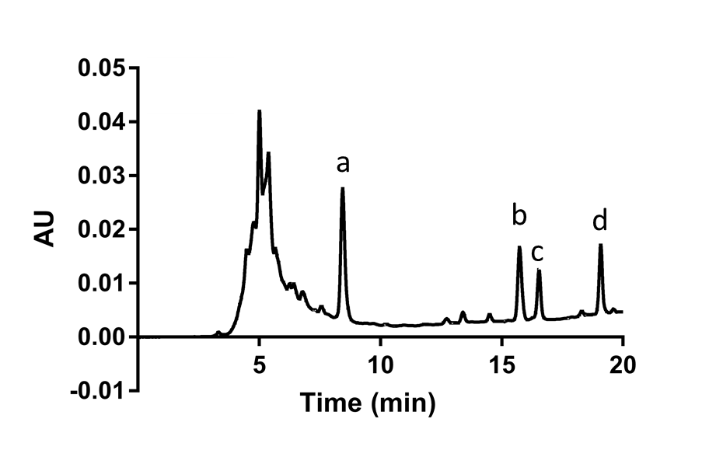
**

Figure S1: High-performance liquid chromatography (HPLC) of *E. cava* extract. HPLC chromatogram for E. cava extract. A Poroshell 120 EC-C18 column (150 x 4.6 mm, 4 μm) was used. Separation was performed with a gradient from 43 to 100% formic acid and methanol within 30 min at a flow rate of 0.3 ml/min. Elution was monitored at 230 nm. There are four phlorotannins such as (a) dieckol, (b) 2,7-phloroglucinol-6,6-bieckol, (c) pyrolgallol-phloroglucino-6,6-bieckol and (d) phlorofucofuroeckol A in E. cava extract [1].


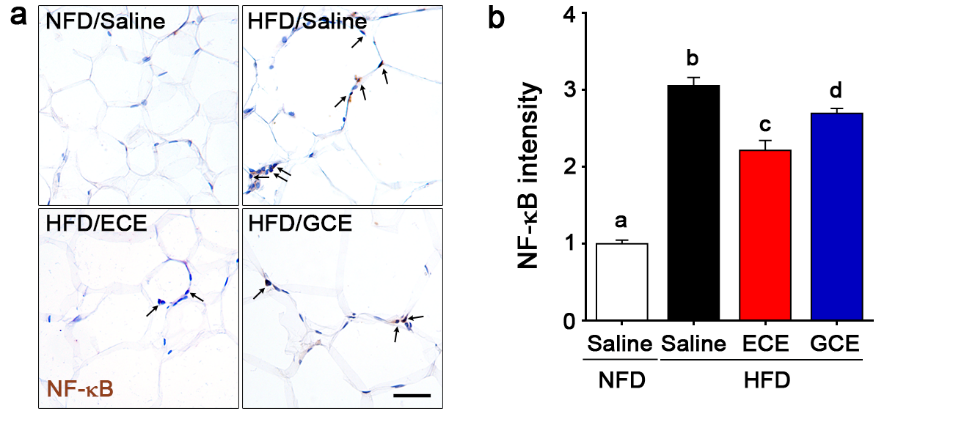


Figure S2: Effects of *E. cava* extract on NF-κB expression in visceral fat of high fat diet (HFD)-induced obese mice. (a) To validate expression of NF-κB in adipose tissue of standard normal fat diet with 0.9% saline (NFD/saline; open bar), HFD with 0.9% saline (HFD/saline; black bar), HFD with *E. cava* extract (HFD/ECE; red bar) and HFD with *Garcinia Cambogia* extract (HFD/GCE; blue bar) group, tissue section slides were prepared. The microscopic images show nuclear factor-kappa-light-chain-enhancer of activated B cells (NF-κB, arrow) expressions were assessed by DAB staining and nuclei were stained hematoxylin (blue). (b) Quantitative green fluorescence intensity of NF-κB co-staining with DAPI graphs show as folds of those of NFD/saline group and these graphs show average expression levels in representative images. Scale bar = 50 µm. Same letters represent no significant difference (p < 0.05).

**Supplementary Table S1: List of primer for quantitative polymerase chain reaction (qRT-PCR)**

| **Gene** | **Primers** | |  |
| --- | --- | --- | --- |
| *β-actin* | Forward | 5'-ACA AAG CTG TTC AGT GTC TCC A-3’ | |
|  | Reverse | 5'-CTC CGT TTC CAG AAT ACA CAC A-3’ | |
| *CD11b* | Forward | 5'-GAC CGA ATC TAC TGG CAA AA AC-3’ | |
|  | Reverse | 5'-TTC TTA TAC TCG GGC CAC ACT T-3’ | |
| *CD80* | Forward | 5'-GAC CGA ATC TAC TGG CAA AAA C-3’ | |
|  | Reverse | 5'-TTC TTA TAC TCG GGC CAC ACT T-3’ | |
| *CD206* | Forward | 5'-TGT ATT CTT TGC CTT TCC CAG T-3’ | |
|  | Reverse | 5'-GAT AAA AGC CAG AAG CAG GAG A-3’ | |
| *CD8a* | Forward | 5'-ATT GGA CTT CGC CTG TGA TAT T-3’ | |
|  | Reverse | 5'-CCT GTG GTA GCA GAT GAG AGT G-3’ | |
| *CD4* | Forward | 5'-AAG ATG AGA CTG ACC CTG AAG C-3’ | |
|  | Reverse | 5'-GGC CAC CAC TTG AAC TAC TTT C-3’ | |
| *IL-6* | Forward | 5'-ACT GGG GAT GTC TGT AGC TCA T-3’ | |
|  | Reverse | 5'-GGG AGT GGT ATC CTC TGT GAA G-3’ | |
| *TNF-α* | Forward | 5'-TTC TGT CTA CTG AAC TTC GGG GTG ATC GGT CC-3’ | |
|  | Reverse | 5'-GTA TGA GAT AGC AAA TCG GCT GAC GGT GTG GG-3’ | |
| *IL-10* | Forward | 5'-ATG GTG TCC TTT CAA TTG CTC T-3’ | |
|  | Reverse | 5'-AGG ATC TCC CTG GTT TCT CTT C-3’ | |
| *TGF-β* | Forward | 5'- CTG GCA GTA GCT CCC CTA TTT A-3’ | |
|  | Reverse | 5'- ACC AGG GTA AAA ATC GAG ATG A-3’ | |
| *NF-κB* | Forward | 5'- AGA AAT CCT ACC CAC AGG TCA A-3’ | |
|  | Reverse | 5'- CAT TTG TGA CCA ACT GAA CGA T-3’ | |
| *SOCS3* | Forward | 5’- ATG GTC ACC CAC AGC AAG TTT-3’ | |
|  | Reverse | 5’- TCC AGT AGA ATC CGC TCT CCT-3’ | |
| *ObR* | Forward | 5’-TGG GCT ACA TCA GGC TTT GAG-3’ | |
|  | Reverse | 5’-CTC TCC TAC AAC CTT CCC CTC-3’ | |
| *AC* | Forward | 5'- AGC TAA CCC AAC TCA GCA AGA C-3’ | |
|  | Reverse | 5'- GCA AAT GGG AAG CAA TAA GAA C-3’ | |
| *HSL* | Forward | 5’-CCC CTG CGA CGA TTA TCA AGA-3’ | |
|  | Reverse | 5’-CAG TGG CTG ATG CAG TTA TGT T-3’ | |
| *PKA* | Forward | 5’-CAA CAA CCG AGT GTG CTT GAT-3’ | |
|  | Reverse | 5’-TCA TTT GCG ATC CGA GTC TGG-3’ | |

**Supplementary Materials and Methods**

*High performance liquid chromatography (HPLC).* A reverse phase high performance liquid chromatography (HPLC) comprising an Agilent Poroshell 120 EC-C18 column (150 x 4.6 mm, 4 μm; Agilent Technologies, Santa Clara, CA, USA) was used. Gradient elution was performed with 0.1% formic acid (FA) and water as the eluents. 43% of the FA and methanol were used between 0 to 2 mins, 70% of the FA and methanol were used up to 8 mins, 100% of FA and methanol were used up to 15 mins and 43% of FA and methanol were used up to 30 mins. Injection volume was 10 µl and the column flow rate was maintained at 0.4 ml/min.

*3,3-Diaminobenzidine (DAB) Staining*. Anti-NF-κB(dilution rate 1:200) antibody was incubated for 1 day at 4℃ to measure the expression level in visceral fat. After washing with PBS solution, incubated with the anti-rabbit HRP secondary antibody for 1hr in blocking solution. Visceral fat slides were used with Avidin-Biotin Complex Staining Kits (ABC Kits; Vector laboratories, USA) was used for 30 min as per the manufacturer’s protocol to amplify the signal (dilution rate 1:50) and then washed with PBS solution and developed with the DAB solution for 4 min. Paraffin slides were stained with hematoxylin staining for 15 secs. Images were collected using light microscopy and measurement of the percentage of colocalized signal with nuclei using Image J software (NIH, USA).

**Supplementary reference**

1. J. H. Lee, J. Y. Ko, J. Y. Oh et al., “Preparative isolation and purification of phlorotannins from Ecklonia cava using centrifugal partition chromatography by one-step,” *Food Chemistry,* vol. 158, pp. 433-437, 2014.
